# Associations of General Self-Efficacy and Digital Health Literacy with Self-Management and Intention to Use Digital Health Services Among Adults Aged 50 Years and Older with Chronic Disease: A Cross-Sectional Study

**DOI:** 10.3390/ijerph23070943

**Published:** 2026-07-22

**Authors:** Pınar Soylar, Gülsüm Aşan, Bircan Ulaş Kadıoğlu

**Affiliations:** 1 Health Sciences Faculty, Firat University, 23119 Elazig, Turkey; 2 Emergency Department, Eğil State Hospital, 21470 Diyarbakır, Turkey; asangulsum.21@gmail.com; 3 Department of Health Science, Osmaniye Korkut Ata University, 80000 Osmaniye, Turkey; dytbircan@gmail.com

**Keywords:** digital health literacy, self-management, general self-efficacy, chronic disease, adults aged 50 years and older

## Abstract

**Highlights:**

**Public health relevance—How does this work relate to a public health issue?**

- This study examined factors associated with chronic disease self-management and intention to use digital health services among adults aged 50 years and older with chronic disease.
- It investigated the roles of digital health literacy and general self-efficacy in digital health engagement within an aging population.

**Public health significance—Why is this work of significance to public health?**

- General self-efficacy showed stronger independent associations with chronic disease self-management and intention to use digital health services than digital health literacy.
- Digital health literacy alone was not independently associated with either outcome after adjustment for demographic and psychosocial factors.

**Public health implications—What are the key implications or messages for practitioners, policy makers and/or researchers in public health?**

- Public health strategies should consider strengthening self-efficacy alongside digital skills when promoting digital health engagement among older adults with chronic disease.
- Future longitudinal and intervention studies are needed to clarify causal pathways and identify factors that influence digital health engagement and chronic disease self-management.

**Abstract:**

Background: Digital health literacy and general self-efficacy may influence chronic disease self-management and the intention to use digital health services among adults aged 50 years and older. This study examined the associations of these factors with self-management and intention to use digital health services. Methods: This cross-sectional descriptive study included 230 adults aged 50 years and older with chronic disease. Participants attended the outpatient clinics of a district state hospital in southeastern Türkiye. Data were collected using the Digital Health Literacy Scale, the Chronic Disease Self-Management Scale, and the General Self-Efficacy Scale. Descriptive statistics, correlation analysis, and hierarchical regression analyses were performed. Results: The mean age of participants was 59.82 ± 7.53 years, and 58.7% were female. Hierarchical regression analyses showed that general self-efficacy was independently associated with both chronic disease self-management (B = 0.015, 95% CI [0.007, 0.023], β = 0.234, *p* < 0.001) and the use of digital health services (B = 0.052, 95% CI [0.005, 0.100], β = 0.289, *p* = 0.005). In contrast, digital health literacy was not independently associated with chronic disease self-management (B = −0.083, 95% CI [−0.173, 0.008], β = −0.118, *p* = 0.174) or intention to use digital health services (B = 0.131, 95% CI: −0.123 to 0.385, β = 0.083, *p* = 0.311). The addition of digital health literacy and general self-efficacy significantly improved the explanatory power of the regression models for chronic disease self-management (ΔR^2^ = 0.067, *p* < 0.001) and intention to use digital health services (ΔR^2^ = 0.117, *p* < 0.001). Conclusions: The findings suggest that general self-efficacy may play a more prominent role than digital health literacy in supporting self-management and digital health service use among adults aged 50 years and older with chronic disease. Interventions aiming to support digital health engagement may benefit from incorporating strategies to strengthen self-efficacy alongside digital skills development. Due to the cross-sectional design, causal inferences cannot be drawn.

## 1. Introduction

Chronic diseases are among the leading causes of mortality and disability worldwide and represent a major public health challenge. Cardiovascular diseases, diabetes, chronic respiratory diseases, and cancers account for a substantial proportion of global morbidity and mortality [1]. The increasing prevalence of chronic diseases is associated with population aging, longer life expectancy, and lifestyle-related risk factors such as physical inactivity, unhealthy diet, and tobacco use. As the burden of chronic diseases continues to rise, maintaining effective long-term disease management has become increasingly important for both individuals and healthcare systems.

Adults aged 50 years and older with chronic diseases may experience increasing challenges in managing their health conditions due to age-related physical, cognitive, and social changes [2,3]. These challenges can negatively affect quality of life, functional capacity, and health-related decision-making processes [4]. In addition, this population may encounter barriers in adapting to rapidly digitalizing healthcare systems, including limited digital skills, lower technology confidence, and difficulties in accessing or evaluating online health information. Therefore, adults aged 50 years and older with chronic disease represent a potentially vulnerable group requiring continuous support in both chronic disease management and digital health engagement.

Effective chronic disease management requires not only access to healthcare services but also individuals’ ability to actively manage symptoms, treatment regimens, lifestyle modifications, and psychosocial challenges in daily life. This process, commonly referred to as self-management, has been associated with improved health outcomes, better quality of life, and reduced healthcare utilization [5,6]. However, maintaining effective self-management may be particularly challenging for adults aged 50 years and older due to physical limitations, multimorbidity, reduced confidence in managing health-related tasks, and difficulties adapting to changing healthcare environments. Therefore, identifying factors associated with self-management is important for supporting long-term disease management in this population. As healthcare systems increasingly rely on digital technologies to support chronic disease management, understanding the factors that facilitate effective engagement with digital health services has become increasingly important.

The rapid digitalization of healthcare systems has transformed how individuals access health information, communicate with healthcare providers, and manage chronic conditions. Digital health services such as telemedicine platforms, electronic health records, online appointment systems, mobile health applications, and internet-based health resources are increasingly integrated into routine healthcare delivery [7,8,9]. These services may support continuity of care, facilitate access to healthcare, and encourage active participation in health management. However, benefiting effectively from digital health services requires more than simply having access to technology. According to the three-level digital divide model, inequalities in digital engagement involve not only access to digital technologies but also differences in digital skills, patterns of use, and the ability to translate digital resources into meaningful health-related outcomes [10]. Therefore, understanding factors associated with the use of digital health services has become increasingly important, particularly among adults aged 50 years and older with chronic diseases.

Digital health literacy refers to the ability to search for, understand, evaluate, and apply health information obtained from digital sources [11]. Adequate digital health literacy may facilitate access to online health information, support communication with healthcare systems, and improve individuals’ ability to use digital health applications effectively [8,12]. Previous studies have reported that higher digital health literacy is associated with better health information-seeking behaviors, greater engagement with digital health technologies, improved self-care, and more favorable health outcomes among older adults and individuals with chronic diseases [13,14,15,16]. However, evidence has also suggested that digital health literacy alone may not be sufficient to explain complex health behaviors such as chronic disease self-management, indicating that additional psychosocial factors may also play an important role. However, previous research suggests that digital health literacy alone may not necessarily translate into effective health behaviors or chronic disease self-management. The ability to benefit from digital health resources may also depend on motivational, psychological, and contextual factors, including confidence in managing health-related tasks and willingness to engage with digital technologies. Adults aged 50 years and older may be particularly vulnerable to difficulties in digital health literacy due to lower technology familiarity, socioeconomic inequalities, and age-related barriers affecting digital engagement [12,13,14]. Digital health literacy and general self-efficacy represent conceptually distinct but complementary constructs. Whereas digital health literacy reflects individuals’ ability to obtain, evaluate, and use health information from digital sources, general self-efficacy reflects their confidence in applying such abilities to manage health-related tasks. Rather than representing competing constructs, digital health literacy and general self-efficacy capture different dimensions of health behavior—one reflecting digital competencies and the other reflecting confidence in applying those competencies to health-related tasks. Examining both constructs simultaneously may therefore provide a more comprehensive understanding of chronic disease self-management and digital health engagement.

Previous international studies have shown that digital health literacy tends to decline with increasing age and may be associated with health status, educational background, and technology use experience [13,14]. However, adults aged 50 years and older represent a heterogeneous population with varying levels of digital engagement and chronic disease burden. In this population, adaptation to digitalized healthcare systems may be influenced not only by chronological age but also by structural, socioeconomic, and psychosocial factors. Therefore, individuals aged 50 years and older may experience vulnerabilities in accessing, evaluating, and effectively using digital health services, particularly in the presence of chronic disease. Consistent with this perspective, the COST Action CA21107 “DIGINET—Ageism and Digitalisation” framework identifies adults aged 50 years and older as a group that may encounter disadvantages in digital transformation processes [17].

Another psychosocial factor potentially associated with chronic disease self-management and digital health engagement is general self-efficacy. According to Bandura’s social cognitive theory, self-efficacy reflects individuals’ beliefs in their capacity to successfully perform behaviors required to achieve desired outcomes [18]. General self-efficacy refers to a broader sense of confidence in managing challenging situations and adapting to changing circumstances [19]. Individuals with higher levels of self-efficacy may be more likely to maintain health-related behaviors, cope with barriers, and engage actively in disease management processes. In the context of digital health, self-efficacy may also influence individuals’ confidence in learning and using digital technologies, evaluating online health information, and integrating digital resources into daily health management. Therefore, general self-efficacy may represent an important psychosocial mechanism underlying both self-management behaviors and intention to use digital health services.

Although previous studies have examined digital health literacy, self-management, or self-efficacy separately, research evaluating these variables together in adults aged 50 years and older with chronic disease remains limited. Findings regarding the relationship between digital health literacy and health-related outcomes have also been inconsistent across different populations and healthcare contexts. Furthermore, few studies have evaluated the independent associations of digital health literacy and general self-efficacy with both chronic disease self-management and intention to use digital health services within the same analytical framework, particularly after accounting for prior digital health service use and relevant sociodemographic characteristics. In Türkiye, the rapid expansion of digital health services has increased the importance of understanding factors associated with digital health engagement among adults aged 50 years and older. This population may face challenges related to digital adaptation while simultaneously experiencing a high burden of chronic disease, making it particularly relevant from both public health and nursing perspectives. Addressing these gaps may contribute to a more comprehensive understanding of adaptation to digitalized healthcare systems among adults with chronic disease.

The present study aimed to examine the associations of digital health literacy and general self-efficacy with chronic disease self-management and intention to use digital health services among adults aged 50 years and older with chronic disease. Based on the existing literature and theoretical framework, it was hypothesized that higher levels of digital health literacy and general self-efficacy would be associated with better self-management and greater intention to use digital health services.

## 2. Materials and Methods

### 2.1. Study Design

This cross-sectional descriptive study examined the associations between self-management, digital health literacy, general self-efficacy, and intention to use digital health services among adults aged 50 years and older with chronic disease. The study was reported in accordance with the Strengthening the Reporting of Observational Studies in Epidemiology (STROBE) guideline for cross-sectional studies.

### 2.2. Population and Sample

The population of the study consisted of individuals aged 50 years and older who had been diagnosed with at least one chronic disease and who applied to the outpatient clinics of a district state hospital in Diyarbakır, located in southeastern Türkiye, between 22 December 2025 and 27 February 2026. Chronic diseases included conditions requiring long-term medical follow-up, such as hypertension, diabetes, chronic obstructive pulmonary disease, and heart disease.

The expected effect size was estimated using the correlation coefficient reported by Hwang et al. [20], who found a significant association between digital health literacy and self-efficacy among older adults (ρ = 0.28, *p* = 0.001). This study examined conceptually similar relationships and was used solely as a reference for sample size estimation rather than for population comparability. With an α level of 0.05 and a statistical power (1 − β) of 0.95, the minimum required sample size was calculated to be 160 participants. To account for potential data loss, a total of 230 individuals were included in the study.

Participants were selected using a non-probability sampling method, specifically consecutive sampling. All eligible individuals who met the inclusion criteria during the study period were invited to participate using a consecutive sampling approach. Because a screening log of all individuals approached was not maintained during data collection, the total number of eligible individuals approached and the response rate could not be determined retrospectively.

The inclusion criteria were: being aged 50 years or older, having at least one physician-diagnosed chronic disease, being able to communicate in Turkish, and voluntarily agreeing to participate in the study. Participants with evident cognitive impairment that could interfere with questionnaire comprehension or communication were not included based on clinical observation during the recruitment process.

### 2.3. Data Collection

Data were collected between 22 December 2025 and 27 February 2026 from participants recruited face-to-face in outpatient clinics. Participants completed the questionnaire using an online form accessed through the researcher’s tablet/mobile device. Before participation, individuals were informed about the study purpose and procedures, and electronic informed consent was obtained through the online form.

For participants who had difficulty reading or independently completing the questionnaire, the researcher read the items aloud in a standardized, neutral, and non-directive manner and recorded responses verbatim according to participants’ statements. Researchers were instructed to read each question verbatim, provide clarification only when participants requested an explanation of the wording, refrain from interpreting the questions or suggesting responses, and record participants’ answers exactly as stated. Care was taken to minimize potential interviewer influence and to maintain participants’ privacy during data collection. The average completion time was approximately 15–20 min.

### 2.4. Data Collection Instruments

Data were collected using a Descriptive Characteristics Form, the Chronic Disease Self-Management Scale, the General Self-Efficacy Scale, and the Digital Health Literacy Scale.

#### 2.4.1. Descriptive Characteristics Form

The Descriptive Characteristics Form was developed by the researcher based on the relevant literature. The form includes questions aimed at determining participants’ sociodemographic characteristics and health-related information. It covers variables such as age, gender, marital status, education level, income level, type and number of chronic diseases, internet access, smartphone use, prior use of digital health services, and intention to use digital health services.

Participants’ intention to use digital health services was assessed by calculating the mean score of three items reflecting future use intentions. These items were: “I plan to perform my health-related activities through digital means as much as possible in the future,” “I would like to use digital applications to monitor my health when possible,” and “I plan to continue using digital systems to follow my medical appointments and results.” The items were rated on a 5-point Likert scale, with higher mean scores indicating a greater intention to use digital health services. The internal consistency coefficient (Cronbach’s alpha) for the three-item intention measure in the present study was α = 0.973.

#### 2.4.2. Chronic Disease Self-Management Scale

The Chronic Disease Self-Management Scale was developed by Ngai et al. [21], and its Turkish validity and reliability were established by Öztürk et al. The scale consists of 21 items and four subdimensions: self-stigma (7 items), coping with stigma (5 items), healthcare efficacy (4 items), and treatment adherence (5 items). Items are rated on a 5-point Likert scale (1 = strongly disagree to 5 = strongly agree).

Scale scores are calculated using the arithmetic mean, with higher scores indicating higher levels of self-management. In the Turkish version, Cronbach’s alpha coefficients for the subscales ranged between 0.789 and 0.876. In the present study, Cronbach’s alpha values were 0.83 for self-stigma, 0.70 for coping with stigma, 0.81 for healthcare efficacy, and 0.88 for treatment adherence [22].

#### 2.4.3. General Self-Efficacy Scale

The General Self-Efficacy Scale was used to assess participants’ levels of general self-efficacy. The scale consists of 10 items with a unidimensional structure, and its Turkish validity and reliability were established by Aypay [19]. Items are rated on a 4-point Likert scale (1 = strongly disagree to 4 = strongly agree).

Total scores range from 10 to 40, with higher scores indicating higher levels of general self-efficacy. In the Turkish adaptation, the Cronbach’s alpha coefficient was reported as 0.83 [19].

#### 2.4.4. Digital Health Literacy Scale

The Digital Health Literacy Scale, developed by van der Vaart and Drossaert [8] and adapted into Turkish by Çetin and Gümüş [23], was used to assess participants’ digital health literacy levels. The scale can be used in scientific studies without requiring additional permission.

The scale consists of 18 items and six subdimensions: Information Searching, Evaluating Reliability, Determining Relevance, Adding Content, Navigational Skills, and Protecting Privacy. Four subdimensions are rated on a 4-point Likert scale (4 = very easy to 1 = very difficult), whereas Navigational Skills and Protecting Privacy are reverse-coded (4 = never to 1 = often).

A total score is not recommended; instead, mean scores (ranging from 1 to 4) are calculated for each subdimension. Scores < 2 indicate low, 2–3 moderate, and >3 high digital health literacy. The Turkish version demonstrated a confirmed six-factor structure through exploratory and confirmatory factor analyses, with an overall Cronbach’s alpha of 0.896 [23].

### 2.5. Data Analysis

Data were analyzed using IBM SPSS Statistics software (Version 20.0). Descriptive statistics were presented as frequencies, percentages, means, and standard deviations. The normality of continuous variables was evaluated using skewness–kurtosis values, histogram plots, and the Kolmogorov–Smirnov test. Since some variables did not demonstrate normal distribution, Spearman correlation analysis was used to examine relationships between continuous variables.

Gender (male = 0, female = 1), independent internet access (no = 0, yes = 1), and previous use of digital health services (no = 0, yes = 1) were included as binary (dummy) variables, while education level was treated as an ordinal variable.

Two separate hierarchical regression analyses were conducted to identify factors associated with chronic disease self-management and intention to use digital health services. In the first block of the regression models, age, gender, education level, multimorbidity, income status, independent internet access, and previous use of digital health services were entered as control variables. In the second block, digital health literacy and general self-efficacy were included. In the regression model predicting intention to use digital health services, chronic disease self-management was additionally included in the second block. The control variables included in Model 1 (age, gender, education level, income status, multimorbidity, independent internet access, and use of digital health services) were selected based on previous literature demonstrating their potential associations with digital health behaviors and chronic disease self-management. Previous use of digital health services was assessed with a single dichotomous (Yes/No) question asking participants whether they had ever used any digital health service, including the e-Nabız Personal Health Record System, the Centralized Hospital Appointment System (MHRS), or hospital mobile applications. This variable was included as a control variable in the hierarchical regression analyses.

Hierarchical regression analysis was used to determine whether digital health literacy and general self-efficacy explained additional variance in the outcome variables beyond these established sociodemographic and health-related characteristics.

Prior to regression analyses, assumptions were tested. Independence of errors was evaluated using the Durbin–Watson statistic. Multicollinearity was assessed using variance inflation factor (VIF) and tolerance values. Linearity, homoscedasticity, and normality of residuals were examined using residual plots and distribution analyses. Standardized regression coefficients (β), explained variance (R^2^), adjusted explained variance (Adjusted R^2^), and changes in explained variance (ΔR^2^) were reported. Statistical significance was accepted as *p* < 0.05. No missing data requiring imputation were identified in the dataset.

### 2.6. Ethical Considerations

This study was approved by the Non-Interventional Research Ethics Committee of Fırat University (Decision date: 11 December 2025; Decision number: 2025/18-29). The study was conducted in accordance with the principles of the Declaration of Helsinki.

All participants were informed about the purpose and scope of the study, and informed consent was obtained. Participation was voluntary, and participants were informed that they could withdraw from the study at any time. All data were collected anonymously and used solely for scientific purposes.

## 3. Results

A total of 230 participants were included in the study (Table 1). The mean age of the participants was 59.82 ± 7.53 years (range: 50–85). Of the participants, 58.7% were female (*n* = 135) and 41.3% were male (*n* = 95). The majority were married (89.1%), while 10.9% were single. Regarding educational level, 48.3% had completed primary education, 29.6% were illiterate, 10.9% had a high school education, and 11.3% had a university degree or higher. In terms of employment status, 21.3% were employed, 22.6% were retired, and 56.1% were not working. Nearly half of the participants reported that their income was equal to their expenses (45.2%), while 43.9% reported income lower than expenses and 10.9% reported income higher than expenses. Concerning health status, 60.0% had one chronic disease, whereas 40.0% had two or more chronic diseases. Most participants reported having independent access to the internet (67.8%), while 32.2% did not have independent internet access.

Descriptive statistics of digital health literacy, chronic disease self-management, general self-efficacy, and intention to use digital health services are presented in Table 2. The mean score of overall digital health literacy was 2.19 ± 0.79 on a scale ranging from 1 to 4. Among the subdimensions, the highest mean score was observed in evaluation of reliability (2.34 ± 0.89), while the lowest score was found in determining the level of interest (2.02 ± 0.95). The mean score of the Chronic Disease Self-Management Scale was 2.53 ± 0.43. Among its subdimensions, treatment adherence had the highest mean score (3.89 ± 0.90), followed by health care efficacy (3.52 ± 1.01) and coping with stigma (3.04 ± 0.88), while self-stigma had the lowest mean score (1.86 ± 0.74). The mean score of the General Self-Efficacy Scale was 30.13 ± 7.16 (range: 10–40). Additionally, the mean score for intention to use digital health services was 3.19 ± 1.24 on a 1–5 scale.

The correlations between digital health literacy, chronic disease self-management, and general self-efficacy are presented in Table 3. A moderate positive and statistically significant correlation was found between chronic disease self-management and general self-efficacy (r = 0.32, *p* < 0.01). However, digital health literacy was not significantly correlated with either chronic disease self-management (r = −0.12) or general self-efficacy (r = −0.02).

A hierarchical regression analysis was conducted to examine the predictors of chronic disease self-management (Table 4). In Model 1, control variables including age, gender, education level, use of digital health services, multimorbidity, monthly income status, and independent internet access were entered as control variables. These variables explained 7.9% of the variance in chronic disease self-management (R^2^ = 0.079, F = 2.709, and *p* = 0.010). Among the control variables, use of digital health services was a significant predictor (B = 0.080, β = 0.232, and *p* = 0.001), while the other variables were not statistically significant (*p* > 0.05). In Model 2, general self-efficacy and digital health literacy were added to the model. The inclusion of these variables significantly improved the model, explaining an additional 6.7% of the variance in chronic disease self-management (ΔR^2^ = 0.067, F change = 8.696, and *p* < 0.001), with the final model explaining 14.6% of the total variance (R^2^ = 0.146). In the final model, general self-efficacy remained a significant positive predictor of chronic disease self-management (B = 0.015, 95% CI [0.007, 0.023], β = 0.234, and *p* < 0.001). In addition, the use of digital health services remained significantly associated with chronic disease self-management (B = 0.052, 95% CI [0.005, 0.100], β = 0.152, and *p* = 0.029). In contrast, digital health literacy was not independently associated with chronic disease self-management (B = −0.083, 95% CI [−0.173, 0.008], β = −0.118, *p* = 0.074).

A hierarchical regression analysis was conducted to examine the predictors of digital health service use intention (Table 5). In Model 1, demographic variables (age, gender, education level, multimorbidity, income status, and internet access) were entered as control variables. This model was statistically significant and explained 9.7% of the variance in digital health service use intention (R^2^ = 0.097, F = 4.001, and *p* = 0.001). Among the control variables, only monthly income status was significantly associated with intention to use digital health services (B = 0.354, β = 0.189, and *p* = 0.006), whereas the remaining control variables were not statistically significant (all *p* > 0.05). In Model 2, chronic disease self-management, digital health literacy, and general self-efficacy were added to the regression model. The inclusion of these variables significantly improved the model, explaining an additional 11.7% of the variance in intention to use digital health services (ΔR^2^ = 0.117, F change = 10.875, and *p* < 0.001), with the final model explaining 21.4% of the total variance (R^2^ = 0.214). In the final model, general self-efficacy was independently associated with intention to use digital health services (B = 0.049, 95% CI [0.026, 0.071], β = 0.281, and *p* < 0.001). Chronic disease self-management was also significantly associated with intention to use digital health services (B = 0.408, 95% CI [0.041, 0.774], β = 0.140, and *p* = 0.029). In contrast, digital health literacy was not independently associated with intention to use digital health services (B = 0.131, 95% CI [−0.123, 0.385], β = 0.083, and *p* = 0.311).

Among sociodemographic variables, monthly income lost its statistical significance after the inclusion of psychosocial variables (*p* = 0.082). No significant associations were found for age, gender, education level, independent internet access, or multimorbidity in the final model. The Durbin–Watson statistic (1.647) indicated no serious autocorrelation in the residuals. Multicollinearity diagnostics indicated no collinearity problems among the predictors (VIF < 2 for all variables).

## 4. Discussion

The present study examined the associations among digital health literacy, chronic disease self-management, general self-efficacy, and intention to use digital health services among adults aged 50 years and older with chronic disease. The findings demonstrated that general self-efficacy was significantly associated with both chronic disease self-management and intention to use digital health services, whereas digital health literacy was not significantly associated with these outcomes. In addition, previous use of digital health services was associated with higher levels of chronic disease self-management. These findings indicate that psychosocial factors, particularly individuals’ confidence in managing health-related tasks, were more strongly associated with chronic disease self-management and intention to use digital health services than digital health literacy in this sample.

Although participants in the present study generally reported access to digital technologies and independent internet use, digital health literacy levels remained moderate, and digital health literacy was not significantly associated with chronic disease self-management or intention to use digital health services. This finding suggests that access to digital resources and basic technology use may not necessarily translate into effective digital health engagement or health-related behavioral outcomes. Consistent with the three-level digital divide framework, meaningful use of digital health technologies may depend not only on access and technical skills but also on individuals’ ability to critically evaluate information, integrate digital resources into daily health management, and adapt to rapidly changing digital environments [8,10,12]. Previous studies have similarly reported that older adults and individuals with chronic diseases may experience difficulties in interpreting and applying digital health information despite having access to digital technologies [24,25,26,27]. In addition, chronic disease self-management involves complex behavioral and psychosocial processes that may not be fully explained by digital competencies alone. Older adults with chronic disease may continue to rely primarily on traditional healthcare interactions, family support, and established self-care routines regardless of their digital literacy level. Nevertheless, the present findings should be interpreted in the context of previous evidence demonstrating positive associations between digital health literacy and health-related outcomes among older adults. A recent systematic review by Shi et al. [27] concluded that digital health literacy is influenced by multiple individual and contextual factors, including educational attainment, cognitive capacity, social support, and prior digital experience. Similarly, Kelly et al. [26] reported that higher digital health literacy was associated with better engagement in health management among individuals with complex chronic conditions. Therefore, the absence of a significant association in the present study should not be interpreted as evidence that digital health literacy is unimportant. Rather, it may indicate that, within this sample, digital health literacy alone was insufficient to explain variation in chronic disease self-management after accounting for psychosocial factors, particularly general self-efficacy. Another possible explanation is the relatively limited variability in digital health literacy observed in the study sample, which may have reduced its ability to discriminate between individuals with different levels of self-management and intention to use digital health services. In addition, the widespread availability of national digital health services in Türkiye, such as e-Nabız and MHRS, may have enabled participants to access digital health resources regardless of their overall level of digital health literacy. Cultural characteristics, including strong family involvement in healthcare decision-making and disease management, may also have attenuated the independent association between digital health literacy and the study outcomes. These factors should be explored in future studies using more diverse populations and longitudinal designs.

Another noteworthy finding of the present study was the significant association between previous use of digital health services and chronic disease self-management. This finding indicates that behavioral engagement with digital health tools was associated with greater involvement in health management processes. Previous studies have reported that repeated engagement with eHealth systems is associated with greater familiarity, confidence, and sustained use of digital health technologies [28,29]. Similarly, individuals who actively use digital health applications have been reported to demonstrate higher levels of engagement, adherence, and self-management behaviors [30,31]. Consistent with these findings, a recent scoping review by Al Mahmud et al. [28] emphasized that the effectiveness of digital health interventions for chronic disease management depends not only on access to digital technologies but also on users’ engagement, confidence, and behavioral readiness. Studies grounded in the Technology Acceptance Model and its extensions further suggest that continued use may be influenced not only by perceived usefulness but also by prior use experience and habit formation, which can reduce cognitive barriers and increase perceived ease of use over time [32,33]. However, due to the cross-sectional design of the present study, the direction of these relationships cannot be determined. It is also possible that individuals with better self-management behaviors may be more willing to engage with digital health services. Therefore, actual use of digital health services may reflect both behavioral engagement and existing self-management capacity. Taken together, these findings indicate that experience with digital health technologies was associated with chronic disease self-management in the present sample; however, the direction of this relationship cannot be determined because of the cross-sectional design.

The prominent role of general self-efficacy observed in the present study is consistent with previous literature emphasizing the importance of self-efficacy in sustaining health behaviors and facilitating active participation in health management processes [34,35]. Digital technologies have been associated with greater perceived control and self-regulation capacities, which may be related to greater confidence in managing health-related behaviors [34]. According to Bandura’s social cognitive theory, individuals with higher levels of self-efficacy may be more likely to cope with barriers, maintain motivation, and adapt to challenging situations, including the use of digital health technologies [18]. Unlike digital health literacy, which primarily reflects domain-specific knowledge and skills, general self-efficacy reflects individuals’ broader confidence in coping with challenges and initiating health-related behaviors. This conceptual distinction provides one possible explanation for why general self-efficacy demonstrated stronger associations with both chronic disease self-management and intention to use digital health services than digital health literacy in the present study. Previous studies have similarly reported that self-efficacy and social support are associated with both digital health engagement and health management behaviors among older adults with chronic conditions [36]. Furthermore, chronic disease self-management is considered a multifactorial behavioral process influenced not only by digital competencies but also by psychological, motivational, and experiential factors [37]. Evidence also indicates that self-efficacy represents an important psychosocial factor associated with self-management behaviors, particularly among individuals with multiple chronic conditions [35]. In addition, meta-analytic findings suggest that self-efficacy may facilitate confidence in using digital technologies and support sustained engagement with digital health tools [38]. Taken together, these findings suggest that digital competencies alone may not be sufficient to explain health-related behaviors and that general self-efficacy showed stronger independent associations with both chronic disease self-management and intention to use digital health services than digital health literacy.

The findings related to intention to use digital health services further support the importance of psychosocial readiness in digital health engagement among adults aged 50 years and older with chronic disease. In the present study, general self-efficacy demonstrated a stronger association with intention to use digital health services than digital health literacy. This finding suggests that individuals’ confidence in their ability to manage health-related tasks and adapt to digital environments may be more influential than digital competencies alone in shaping technology use intentions. Previous studies have similarly emphasized the importance of self-efficacy, trust, perceived ease of use, and confidence in determining the acceptance and sustained use of digital health technologies among older adults [15,16,39,40,41]. From a practical perspective, these findings suggest that interventions aiming to support digital health engagement could benefit not only from improving digital skills but also from strengthening individuals’ confidence, motivation, and perceived control in using digital technologies. Health professionals, particularly nurses, may play an important supportive role in this process by facilitating guided use, providing individualized support, and encouraging gradual adaptation to digital health environments. Future studies should examine whether these associations differ according to age group, educational attainment, sex, or type of chronic disease, and should explore potential mediating or moderating mechanisms linking digital health literacy, self-efficacy, and health-related behaviors. However, longitudinal and interventional studies are needed to better understand the causal pathways underlying these relationships.

## 5. Conclusions

This study contributes to the understanding of the relationships among digital health literacy, general self-efficacy, chronic disease self-management, and intention to use digital health services among adults aged 50 years and older with chronic disease. The findings suggest that general self-efficacy may represent a more prominent psychosocial factor associated with both self-management and intention to use digital health services, whereas digital health literacy alone was not significantly associated with these outcomes. These findings indicate that access to digital health information and digital competencies alone were not independently associated with health-related behaviors in this sample. Instead, individuals’ confidence in their ability to manage health-related tasks and adapt to digital environments may play a more influential role in digital health engagement and chronic disease self-management. From a public health and nursing perspective, these findings provide preliminary evidence that may inform the development of future interventions to support digital health engagement among adults with chronic disease. Such interventions may consider combining digital skill development with strategies that strengthen self-efficacy, motivation, and confidence in using digital health technologies. However, longitudinal and intervention-based studies are needed before causal or practice recommendations can be established.

Future research should employ longitudinal and intervention-based designs to clarify the temporal and causal relationships among digital health literacy, general self-efficacy, chronic disease self-management, and intention to use digital health services. In addition, future studies should investigate potential mediating and moderating mechanisms and examine whether these associations differ according to age, sex, educational attainment, or type of chronic disease.

## 6. Limitations of the Study

This study has several limitations that should be considered when interpreting the findings. First, due to the cross-sectional design, causal inferences cannot be made regarding the relationships between the study variables. Second, the study was conducted in a single district state hospital in southeastern Türkiye, which may limit the generalizability of the findings to different populations and healthcare settings. Third, the sample consisted of adults aged 50 years and older with chronic disease who were able to access and use digital technologies to some extent, potentially excluding individuals with more limited digital access or severe digital disadvantages. In addition, some participants completed the questionnaire with researcher assistance due to literacy or reading difficulties, which may have increased the risk of response or social desirability bias despite efforts to maintain a neutral and standardized data collection process. In addition, because a screening log was not maintained during participant recruitment, the response rate could not be calculated, and potential selection bias cannot be completely excluded. Finally, all data were collected using self-report measures, which may be subject to recall bias and subjective interpretation. Because all variables were measured using self-report questionnaires administered at a single time point, common method bias cannot be excluded.

Although validated instruments were used, measurement error related to self-reported responses cannot be completely excluded.

## Figures and Tables

**Table 1:** Sociodemographic and Health-Related Characteristics of the Participants.

Variables	*n*	%
Mean Age	59.82 ± 7.53	Min: 50–Max: 85
Gender		
Female	135	58.7
Male	95	41.3
Marital Status		
Married	205	89.1
Single	25	10.9
Education Level		
Illiterate	68	29.6
Primary school	111	48.3
High School	25	10.9
University and above	26	11.3
Employment status		
Employed	49	21.3
Retired	52	22.6
Not working	129	56.1
Income Status		
Income less than expenses	101	43.9
Income equal to expenses	104	45.2
Income greater than expenses	25	10.9
Chronic Disease Status		
One chronic disease	138	60.0
Two or more chronic diseases	92	40.0
Independent Internet Access		
Yes	156	67.8
No	74	32.2

**Table 2:** Descriptive Statistics for Digital Health Literacy, Chronic Disease Self-Management, General Self-Efficacy, and Intention to Use Digital Health Services.

Scale/Subdimension	Minimum	Maximum	Mean ± SD
Digital Health Literacy *	1.00	4.00	2.19 ± 0.79
Information Searching	1.00	4.00	2.19 ± 0.89
Evaluation of Reliability	1.00	4.00	2.34 ± 0.89
Determining Level of Relevance	1.00	4.00	2.02 ± 0.95
Adding Content	1.00	4.00	2.09 ± 0.86
Navigation Skills	1.00	4.00	2.21 ± 0.92
Privacy Protection	1.00	4.00	2.27 ± 1.05
Chronic Disease Self-Management Scale	1.48	3.86	2.53 ± 0.43
Self-Stigma	1.00	4.00	1.86 ± 0.74
Coping with Stigma	1.00	5.00	3.04 ± 0.88
Health Care Efficacy	1.00	5.00	3.52 ± 1.01
Treatment Adherence	1.60	5.00	3.89 ± 0.90
General Self-Efficacy Scale	10.00	40.00	30.13 ± 7.16
Intention to Use Digital Health Services	1.00	5.00	3.19 ± 1.24

* The overall digital health literacy score represents the mean of the six digital health literacy subdimension scores and was used as a composite indicator in descriptive and regression analyses. Note: Digital Health Literacy Scale subdimension scores range from 1 to 4, Chronic Disease Self-Management Scale scores range from 1 to 5, General Self-Efficacy Scale scores range from 10 to 40, and intention to use digital health services scores range from 1 to 5. Higher scores indicate higher levels of the corresponding construct.

**Table 3:** Correlations Between The Study Variables.

Variables	DHL	Self Management	General Self-Efficacy
DHL	1	−0.12	−0.02
Self management	−0.12	1	0.32 **
General self-efficacy	−0.02	0.32 **	1

Spearman correlation coefficients are presented. ** *p* < 0.01.

**Table 4:** Hierarchical Multiple Linear Regression Analysis for Predictors of Chronic Disease Self-Management.

Variables	Model 1 B (SE)	β	*p*	Model 2 B (SE)	β	*p*	95% CI for B (Model 2)
Control Variables							
Age	0.004 (0.004)	0.078	0.289	0.003 (0.004)	0.055	0.449	−0.006 to 0.011
Gender	0.099 (0.063)	0.114	0.115	0.070 (0.061)	0.074	0.294	−0.051 to 0.190
Education level	−0.034 (0.025)	−0.105	0.178	−0.005 (0.027)	−0.010	0.903	−0.058 to 0.048
Use of digital health services	0.080 (0.023)	0.232	<0.001	0.052 (0.024)	0.289	0.005	0.005 to 0.100
Multimorbidity	−0.021 (0.057)	−0.024	0.717	−0.025 (0.056)	−0.026	0.681	−0.135 to 0.085
Income status	0.039 (0.045)	0.061	0.386	−0.002 (0.045)	0.001	0.988	−0.090 to 0.086
Independent internet access	0.056 (0.070)	0.061	0.423	0.098 (0.072)	0.105	0.184	−0.044 to 0.240
Main Variables							
General self-efficacy (GSE)		—	—	0.015 (0.004)	0.234	<0.001	0.007 to 0.023
Digital health literacy (DHL)		—	—	−0.083 (0.046)	−0.118	0.174	−0.173 to 0.008
Model Fit Statistics							
R^2^		0.079			0.146		
Adjusted R^2^		0.050			0.111		
ΔR^2^		—			0.067		
F Change		—			8.696	<0.001	
Durbin–Watson					1.818		

B = unstandardized regression coefficient; SE = standard error; β = Standardized regression coefficient; CI = confidence interval. Model 1: Control variables. Model 2: Control variables + general self-efficacy and digital health literacy. All VIF values were below 2, and tolerance values were above 0.20, indicating no multicollinearity problem.

**Table 5:** Hierarchical regression analysis predicting intention to use digital health services.

Variables	Model 1 B (SE)	β	*p*	Model 1 B (SE)	β	*p*	95% CI for B (Model 2)
Control Variables							
Age	−0.021 (0.012)	−0.128	0.077	−0.021 (0.011)	−0.128	0.064	−0.043 to 0.001
Gender	−0.185 (0.179)	−0.074	0.303	−0.244 (0.170)	−0.097	0.153	−0.579 to 0.091
Education level	0.023 (0.073)	0.024	0.751	0.038 (0.075)	0.040	0.613	−0.109 to 0.185
Multimorbidity	0.025 (0.163)	0.010	0.878	−0.044 (0.155)	−0.018	0.775	−0.351 to 0.262
Income status	0.354 (0.127)	0.189	0.006	0.216 (0.123)	0.115	0.082	−0.028 to 0.459
Independent internet access	0.244 (0.200)	0.092	0.223	0.105 (0.202)	0.040	0.613	−0.292 to 0.503
Main Variables							
Digital health literacy (DHL)	—	—	—	0.131 (0.129)	0.083	0.311	−0.123 to 0.385
General self-efficacy (GSE)	—	—	—	0.049 (0.011)	0.281	<0.001	0.026 to 0.071
Chronic disease self-management	—	—	—	0.408 (0.186)	0.140	0.029	0.041 to 0.774
Model fit statistics							
R^2^		0.097			0.214		
Adjusted R^2^		0.073			0.182		
ΔR^2^					0.117	<0.001	
F Change					10.875	<0.001	
Durbin–Watson						1.647	

B = unstandardized regression coefficient; SE = standard error β = Standardized regression coefficient; CI = confidence interval; β = Standardized regression coefficient. Model 1: Control variables. Model 2: Control variables + digital health literacy + general self-efficacy + chronic disease self-management. All VIF values were below 2, and tolerance values were above 0.20, indicating no multicollinearity problem.

## Data Availability

The raw data supporting the conclusions of this article will be made available by the authors on request.
